# The Role of Membranes and Lipid-Protein Interactions in the Mg-Branch of Tetrapyrrole Biosynthesis

**DOI:** 10.3389/fpls.2021.663309

**Published:** 2021-04-28

**Authors:** Katalin Solymosi, Beata Mysliwa-Kurdziel

**Affiliations:** ^1^Department of Plant Anatomy, ELTE Eötvös Loránd University, Budapest, Hungary; ^2^Department of Plant Physiology and Biochemistry, Faculty of Biochemistry, Biophysics and Biotechnology, Jagiellonian University, Krakow, Poland

**Keywords:** chlorophyll biosynthesis, chloroplast, etioplast, NADPH:protochlorophyllide oxidoreductase, phytol, prolamellar body, protochlorophyllide, tubular complex

## Abstract

Chlorophyll (Chl) is essential for photosynthesis and needs to be produced throughout the whole plant life, especially under changing light intensity and stress conditions which may result in the destruction and elimination of these pigments. All steps of the Mg-branch of tetrapyrrole biosynthesis leading to Chl formation are carried out by enzymes associated with plastid membranes. Still the significance of these protein-membrane and protein-lipid interactions in Chl synthesis and chloroplast differentiation are not very well-understood. In this review, we provide an overview on Chl biosynthesis in angiosperms with emphasis on its association with membranes and lipids. Moreover, the last steps of the pathway including the reduction of protochlorophyllide (Pchlide) to chlorophyllide (Chlide), the biosynthesis of the isoprenoid phytyl moiety and the esterification of Chlide are also summarized. The unique biochemical and photophysical properties of the light-dependent NADPH:protochlorophyllide oxidoreductase (LPOR) enzyme catalyzing Pchlide photoreduction and located to peculiar tubuloreticular prolamellar body (PLB) membranes of light-deprived tissues of angiosperms and to envelope membranes, as well as to thylakoids (especially grana margins) are also reviewed. Data about the factors influencing tubuloreticular membrane formation within cells, the spectroscopic properties and the *in vitro* reconstitution of the native LPOR enzyme complexes are also critically discussed.

## Introduction

Thylakoid membranes of photosynthetic organisms have a unique and highly conserved lipid composition: in addition to the phospholipid, phosphatidylglycerol (PG), they predominantly contain galactolipids (monogalactosyldiacylglycerol—MGDG, digalactosyldiacylglycerol—DGDG, and sulfoquinovosyldiacylglycerol—SQDG). Galactolipids are major components of plastid inner membranes that play an important role in chloroplast differentiation from proplastids or etioplasts, in chlorophyll (Chl) biosynthesis, in the accumulation of light-harvesting proteins (Fujii et al., 2019a,b), and have been also identified as structural components of several major protein complexes of the photosynthetic apparatus (PSII, PSI, LHCII, and cytochrome *b*_6_*f* ) (Kobayashi, 2016). In this review, we provide an overview of the Mg-branch of tetrapyrrole biosynthesis, leading to Chl biosynthesis, that occurs in plastids and is associated to plastid membranes. We focus on the reduction of protochlorophyllide (Pchlide) to chlorophyllide (Chlide), and the role of lipids and plastid inner membranes in the process. Two distinct enzymes have been evolved to catalyze this reaction step: first a nitrogenase-like, oxygen-sensitive dark-operative NADPH:Pchlide oxidoreductase enzyme (DPOR), and later a light-dependent NADPH:protochlorophyllide oxidoreductase (LPOR), which requires light for its activity (Gabruk and Mysliwa-Kurdziel, 2015; Vedalankar and Tripathy, 2019). There is very low sequence homology between these two enzymes (Gabruk et al., 2012), and in most organisms they occur simultaneously. However, angiosperms lack LPOR and became unable to synthesize Chlide and chlorophylls (Chls) in the absence of light. Due to this special feature, Chl biosynthesis has been extensively studied in dark-germinated angiosperm seedlings which also have agricultural relevance as the seeds of many crops are sown deep into the soil and thus start to germinate in the dark. Due to space limitation our focus is on data available about the role of lipids and membranes on Chl biosynthesis in angiosperms.

## Overview of the Mg-Branch of Tetrapyrrole Biosynthesis

Chlorophylls (i.e., Chl *a* and Chl *b*) are the main photosynthetic pigments in plants. Concerning their molecular structure, they belong to tetrapyrroles (Fiedor et al., 2019). Their biosynthesis takes place in plastids and shares some common steps with that of other tetrapyrroles. The tetrapyrrole biosynthesis route leading to Chl formation is often referred to as the Mg-branch (for review see Mochizuki et al., 2010; Tanaka et al., 2011; Tripathy and Pattanayak, 2012; Rebeiz, 2014; Willows, 2019; Bryant et al., 2020). Several new data indicate that galactolipids play crucial roles in Chl biosynthesis (Kobayashi et al., 2014; Fujii et al., 2019b) in addition to other factors regulating the Mg-branch at various levels and *via* different mechanisms as reviewed by Grimm (2010), Stenbaek and Jensen (2010), Zhang et al. (2011), Czarnecki and Grimm (2012), Richter and Grimm (2013), Brzezowski et al. (2015), Wang and Grimm (2015), Kobayashi and Masuda (2016), Yuan et al. (2017), and Herbst et al. (2019).

### Formation of Mg-protoporphyrin IX

**Figure 1:**
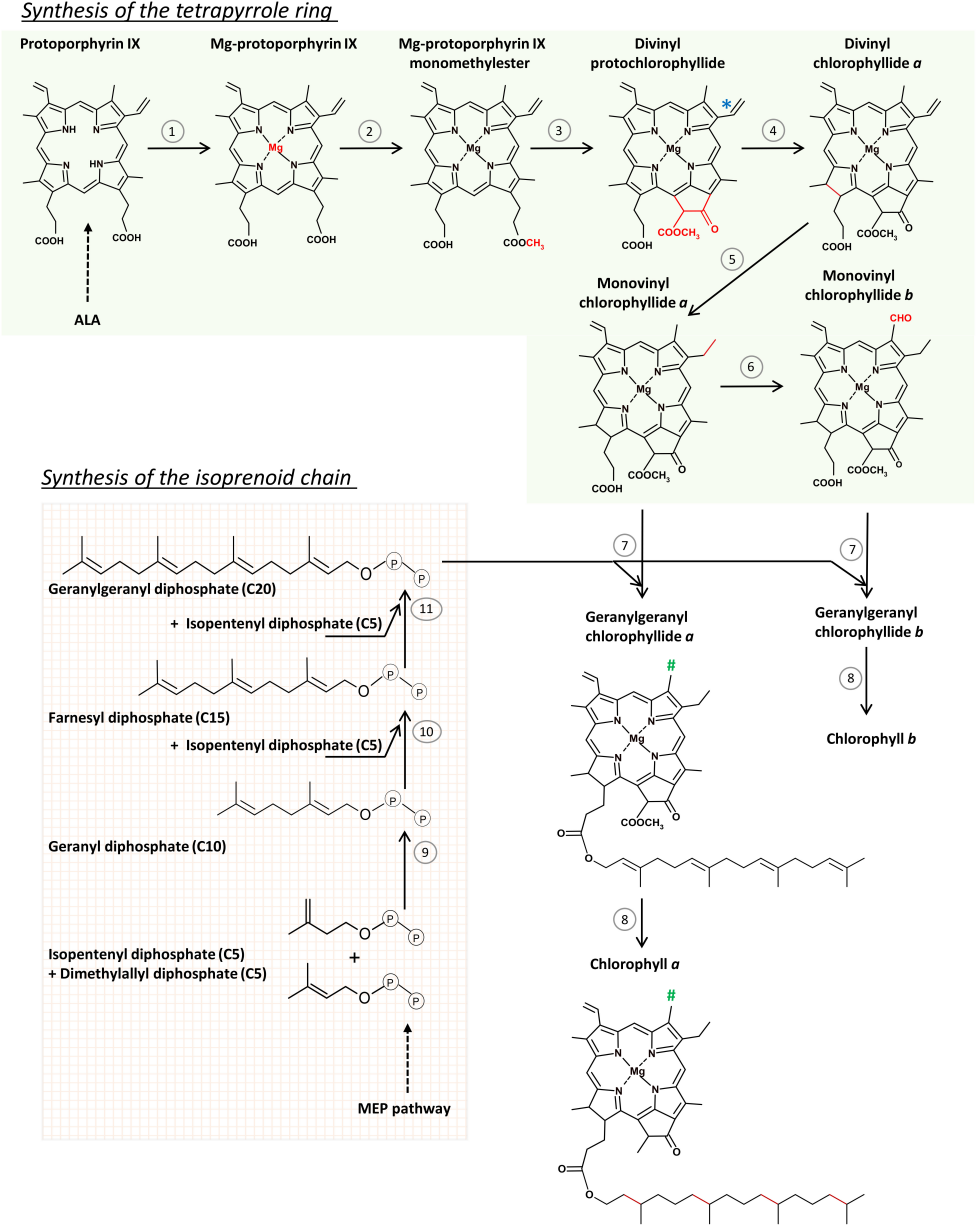
The scheme of chlorophyll (Chl) biosynthesis in angiosperms. Reactions of the tetrapyrrole ring formation route (1–6) are catalyzed by: (1) Mg-chelatase, (2) protoporphyrin IX methyltransferase, (3) Mg protoporphyrin IX monomethyl ester (oxidative) cyclase, (4) light-dependent NADPH:protochlorophyllide oxidoreductase, (5) divinyl reductase, (6) chlorophyllide oxygenase, (7) chlorophyll synthase, (8) geranylgeranyl reductase. (*) Divinyl reductase (5) can also convert the indicated divinyl group to a monovinyl group in protochlorophyllide molecule. (#) Methyl group in Chl *a*, and formyl group in Chl *b*. Reactions of the isoprenoid chain formation (9–11) are catalyzed by geranyl diphosphate and geranylgeranyl diphosphate synthases. See text for further explanations.

The incorporation of Mg^2+^ to protoporphyrin IX is the first reaction on the Mg-branch of tetrapyrrole biosynthesis and the protoporphyrin IX is the last common intermediate of both Chl and heme biosynthesis (Figure 1). This reaction is catalyzed by Mg-chelatase (EC 6.6.1.1) which is a large multisubunit complex composed of three subunits, CHLI, CHLD, and CHLH. Two of them, CHLD and CHLI, catalyze ATP hydrolysis, whereas the third one, CHLH, binds the substrate (protoporphyrin IX). During the two-steps reaction, first, an ATP- and Mg^2+^-dependent activation occurs, leading to the formation of the active Mg-chelatase complex, which is then followed by the ATP-dependent chelation step (reviewed by Masuda, 2008; Bryant et al., 2020). Recent detailed analyses unraveled the conformational changes and kinetic parameters of CHLH caused by the substrate binding (Adams et al., 2020). Two CHLI isoforms, CHLI1 and CHLI2, were found in *A. thaliana*, both having similar expression profiles. The dominant CHLI1 isoform is crucial for the chelatase activity, whereas CHLI2 plays a limited role in Chl biosynthesis but certainly contributes to the assembly of the Mg-chelatase complex (Kobayashi et al., 2008).

GUN4 is a positive regulator of Mg-chelatase and is involved in plastidic retrograde signaling (Larkin et al., 2003; Davison et al., 2005). Moreover, it is a key regulator of Chl biosynthesis acting on the posttranslational level (Peter and Grimm, 2009). Together with protoporphyrin IX, it was suggested to promote interactions between CHLH and chloroplast membranes (Adhikari et al., 2009). Changes in the activity of the Mg-branch under different light conditions is probably regulated by GUN4 phosphorylation (Richter et al., 2016).

### From Mg-protoporphyrin IX to Protochlorophyllide

Mg-protoporphyrin is converted to 3,8 vinyl-Pchlide (divinyl-Pchlide or DV-Pchlide) in two successive reactions. The first is the methylation of Mg-protoporphyrin IX catalyzed by Mg-protoporphyrin IX methyltransferase (EC 2.1.1.11). The second is the formation of the isocyclic ring E catalyzed by different cyclases in oxygenic and anoxygenic phototrophs, according to different mechanisms (Chen et al., 2017). In eukaryotic oxygenic phototrophs that include angiosperms, mostly discussed in this work, the formation of the E ring is catalyzed by Mg-protoporphyrin IX monomethyl ester (oxidative) cyclase (EC 1.14.13.81). This is an oxygen-dependent enzyme, composed of multiple subunits (Chereskin et al., 1982; Wong and Castelfranco, 1984, 1985; Rzeznicka et al., 2005). To keep the enzymatic activity, Mg-protoporphyrin IX monomethylester cyclase (CHL27/CRD1) (Tottey et al., 2003) requires the presence of an auxiliary factor YCF54, which is the hypothetical chloroplast open reading frame 54 (Albus et al., 2012; Chen and Hunter, 2020). Among various factors, YCF54 interacts with ferredoxin-NADPH reductase (FNR1), which may deliver electrons from the photosynthetic electron transport chain to the cyclase (Herbst et al., 2018). However, the exact mechanism of the formation of the isocyclic E ring is still not fully elucidated.

A specific reductase, 8-vinyl reductase (EC 1.3.1.75 also called divinyl reductase, i.e., DVR), can reduce one of the two vinyl groups in DV-Pchlide converting DV-Pchlide to 3-vinyl Pchlide (monovinyl-Pchlide or MV-Pchlide), a reaction observed in etiolated seedlings (Belanger and Rebeiz, 1979, 1980; Shioi and Takamiya, 1992) but also during the night phases of photoperiodic growth (Carey and Rebeiz, 1985; Carey et al., 1985; Tripathy and Rebeiz, 1988). This observation contributed to the categorization of plants depending on whether they accumulate predominantly DV-Pchlide or MV-Pchlide during daytime or nights of photoperiodic growth (Ioannides et al., 1994; Mageed et al., 1997). In particular, Arabidopsis (DDV-LDV, i.e., a dark divinyl-light divinyl plant) accumulates mainly DV-Pchlide independently on light conditions whereas wheat and rice accumulate MV-Pchlide at night and mostly DV-Pchlide at daytime and are thus considered as DMV-LDV (i.e., dark monovinyl-light divinyl) plants. Further analysis of the accumulation of other MV- and DV- tetrapyrrole intermediates and their interconversion in various plant species led to a multibranched pathway, including parallel MV and DV paths of Chl biosynthesis interconnected by DVR (Tripathy and Rebeiz, 1986; Rebeiz et al., 1999; Wang et al., 2013). Plant DVR is a NADPH-dependent enzyme (Parham and Rebeiz, 1992; Whyte and Griffiths, 1993) and shows a broad substrate specificity (Wang et al., 2013), however, it is the most active for DV-Chlide as substrate (Parham and Rebeiz, 1992; Nagata et al., 2007; Wang et al., 2013). A putative transmembrane α helix has been identified near the C terminus of DVR (Nakanishi et al., 2005; Wang et al., 2010).

Both MV-Pchlide and DV-Pchlide are accepted as substrates by the enzymes catalyzing the subsequent reactions leading finally to the formation of the Chl *a* molecule (Knaust et al., 1993; Nagata et al., 2007). What is more, most literature data dealing with the last steps of Chl biosynthesis in angiosperms did not determine the exact nature of Pchlide (e.g., MV- or DV-Pchlide) in their samples. Therefore, and for simplicity, we decided to refer to the pigment below as Pchlide. Pchlide is a key intermediate of Chl biosynthesis in angiosperms. It is a porphyrin type compound, capable of light absorption. However, its Q absorption bands are strongly blue-shifted compared to that of Chls (Fiedor et al., 2019). The photophysical properties of Pchlide in various model systems and in plastids are thoroughly discussed in section LPOR—An Enzyme Operating in Lipid Environment.

### Light-Triggered Reduction of Protochlorophyllide to Chlorophyllide

DPOR (EC 1.3.7.7) is an ancestral but oxygen-sensitive enzyme catalyzing Pchlide reduction. According to a widely accepted long-held hypothesis, LPOR (EC 1.3.1.33) emerged as an evolutionary response to the increasing level of atmospheric oxygen (Fujita, 1996; Schoefs and Franck, 2003; Yamazaki et al., 2006; Reinbothe et al., 2010) caused by the emergence of oxygenic photosynthesis around 2.4 billion years ago (Suzuki and Bauer, 1995), and occurs only in oxygenic phototrophs. However, LPOR was discovered a few years ago in the aerobic anoxygenic phototrophic α-proteobacterium *Dinoroseobacter shibae* (Kaschner et al., 2014), and in other anoxygenic organisms (Chernomor et al., 2020), probably as a result of horizontal gene transfer from cyanobacteria. Further investigations are required to elucidate the evolutionary origin and distribution of LPOR. In angiosperms, the *LPOR* gene was duplicated several times, leading to the formation of LPOR isoforms in several species denoted as LPOR-A, LPOR-B, and LPOR-C (Gabruk and Mysliwa-Kurdziel, 2020) (discussed in detail in section Biochemical Characterization of LPOR).

In angiosperms, the reduction of Pchlide to Chlide is catalyzed by LPOR (for review see Scrutton et al., 2012; Gabruk and Mysliwa-Kurdziel, 2015). The photocatalytic reaction catalyzed by LPOR is the reduction of one of the conjugated double bonds in the porphyrin ring of Pchlide, thus converting it to a chlorine. The photophysical properties of the product, Chlide, are only slightly different from those of Chl (Fiedor et al., 2003, 2008). LPOR activity is induced by light and is fully inhibited in the dark. No chemical LPOR inhibitors are known. The biochemical characterization of LPOR and the reaction mechanism of the photoreduction are discussed in section LPOR—An Enzyme Operating in Lipid Environment.

### From Chlorophyllide to Chlorophyll

The esterification of Chlide with an isoprenyl alcohol leads to the formation of Chl *a* molecule. Below, we'll first review the formation of the phytol chain, and then the esterification reaction itself.

#### Biosynthesis of the Isoprenyl Side Chain

The isoprenyl side chain of Chl is a phytyl moiety. Phytol is a 20-carbon (C20) alcohol, which is structurally related to geranylgeraniol, however, it is more saturated. The prenyl backbone of geranylgeraniol is synthesized in chloroplasts *via* the 2-methylerythritol-4-phosphate (MEP) pathway (Lichtenthaler et al., 1997), one of two pathways of isoprenoid biosynthesis present in plant cells. The characterization and detailed description of these pathways is beyond the scope of this paper and can be found in Rodriguez-Concepcion (2010), Vranová et al. (2013), Rodríguez-Concepción and Boronat (2015), and Gutbrod et al. (2019).

Geranylgeranyl diphosphate (C20) is formed from two prenyl diphosphate precursors: isopentenyl diphosphate (C5) and dimethylallyl diphosphate (C5), both produced in the MEP pathway, in sequential condensation reactions. Their condensation leads to geranyl diphosphate (C10). The condensation of geranyl diphosphate (C10) with an isopentenyl diphosphate (C5) molecule results in the formation of farnesyl diphosphate (C15), which can further condensate with another isopentenyl diphosphate (C5) molecule to form geranylgeranyl diphosphate (C20). These condensation reactions are catalyzed by the geranyl diphosphate and geranylgeranyl diphosphate synthases, EC 2.5.1.10 and EC 2.5.1.29, respectively, which are enzymes belonging to the family of isopentenyl-diphosphate synthases (prenyltransferases) (Gutbrod et al., 2019). Geranylgeranyl diphosphate (C20) is an important intermediate not only for Chl synthesis, but also for other biochemical pathways including the biosynthesis of carotenoids, prenyllipids and plant hormones (gibberellins). However, regulatory mechanisms of geranylgeranyl diphosphate consumption among these biosynthesis pathways are not yet fully understood.

Interestingly, isopentenyl diphosphate or farnesyl diphosphate formed in the cytosol by the alternative pathway of isoprenoid biosynthesis, the so-called mevalonate pathway (MVA), can contribute to the synthesis of chloroplast isoprenoids (Nagata et al., 2002; Bick and Lange, 2003; Opitz et al., 2014; Manzano et al., 2016; Pellaud and Saffrané, 2017). However, isoprenoids derived from the mevalonate pathway cannot substitute for the deficiency in the flux through the MEP pathway (Nagata et al., 2002). Geranylgeranyl diphosphate deficiency leads to overaccumulation of Chlide, which can be a source of photooxidative stress (Kim et al., 2013).

The plastid geranylgeranyl diphosphate synthase was first purified from *Capsicum* chromoplasts (Dogbo and Camara, 1987) and *Sinapsis alba* etioplasts (Laferrière and Beyer, 1991), and then cloned and further characterized (Kuntz et al., 1992). Multiple geranylgeranyl diphosphate synthase families were characterized in Arabidopsis (Beck et al., 2013) and some other plants (Wang and Dixon, 2009; Zhou et al., 2017; Wang et al., 2019). They show specific subcellular localizations and different expression patterns, which may be important for geranylgeranyl diphosphate synthesis depending on metabolic pathways, developmental stages, or specific tissues. Seven of 10 synthases found in Arabidopsis were localized to plastids. One of them (GGPPS11) mainly participates in the biosynthesis of plastid isoprenoids including Chls (Beck et al., 2013; Ruiz-Sola et al., 2016). GGPPS11 operates as a dimer with another geranylgeranyl diphosphate synthase, GGPPS12, which lacks motifs required for prenyl binding and is catalytically inactive (Beck et al., 2013; Ruiz-Sola et al., 2016). Binding of this accompanying protein regulates the enzyme specificity between the production of geranylgeranyl diphosphate and geranyl diphosphate. The accompanying protein in rice, which is called a recruiting molecule, controls the dimerization of geranylgeranyl diphosphate synthase and enhances its catalytic activity (Zhou et al., 2017). Moreover, it determines the allocation of the enzyme from the stroma to the thylakoid membranes, which is a way to control the flux of geranylgeranyl diphosphate toward Chl biosynthesis.

It is noteworthy to mention that independently from Chl biosynthesis, peculiar plastid inner membranes can be observed in plastids accumulating isoprenoids (Turner et al., 2000, 2012; reviewed e.g., in Solymosi and Schoefs, 2010) which indicates some direct or indirect interaction of these lipophilic molecules with the biomembranes.

#### Esterification of Chlide

Chlide is esterified with geranylgeranyl diphosphate to form geranylgeranyl Chlide (GG-Chlide). This reaction is catalyzed by chlorophyll synthase (EC 2.5.1.62) (Rüdiger et al., 1980). Pchlide is not accepted as the substrate for this enzyme (Griffiths, 1974; Rüdiger et al., 1980). However, in the two subsequent reactions, i.e., the photoreduction of Pchlide and the esterification of Chlide, the same part of the tetrapyrrole molecule is modified. Pchlide and Chlide are thus bound to the catalytic site of the respective enzymes, i.e., LPOR and chlorophyll synthase, in the same orientations (Helfrich et al., 1994, 1996; Rüdiger et al., 2005). Chlorophyll synthase binds the isoprenoid chain before binding of the second substrate, Chlide (Schmid et al., 2002).

Finally, the geranylgeranyl reductase (EC 1.3.1.83) reduces subsequently three double bonds in geranylgeranyl moiety of GG-Chlide and converts it to phytyl moiety, yielding this way Chl *a*. Observations that mainly GG-Chlide was detected shortly after illumination of etiolated seedlings (Schoch, 1978) whereas Chl *a*, having a phytyl moiety, was found in green barley seedlings (Soll et al., 1983) opened up a long-lasting discussion on the order of the esterification and reduction steps in plants. However, Schoefs and Bertrand (Schoefs and Bertrand, 2000) proved that the transformation of Chlide to Chl in developing seedlings is a four-steps process, which includes the successive formation of GG-Chlide, dihydrogeranylgeranyl Chlide and tetrahydrogeranylgeranyl Chlide as Chl *a* biosynthesis intermediates. The reduction of geranylgeranyl moiety of GG-Chlide was confirmed in tobacco cell cultures (Benz et al., 1984). Moreover, the preferential use of geranylgeranyl diphosphate than phytyl diphosphate by recombined *Arabidopsis thaliana* chlorophyll synthase was observed (Gaubier et al., 1995; Oster and Rüdiger, 1997). Chlides *a* and *b* were esterified at the same rate by recombinant chlorophyll synthase (Oster and Rüdiger, 1997).

In plastids, the same geranylgeranyl reductase may operate in different pathways of hydrogenation of geranylgeranyl moiety. The hydrogenation of GG-Chlide for Chl biosynthesis occurs in thylakoids whereas the reduction of geranylgeranyl diphosphate to phytyl diphosphate during the synthesis of tocopherols takes place in the envelopes (Soll et al., 1983; Keller et al., 1998). In etiolated seedlings the hydrogenation process is slowed down making it easier to observe than in green leaves. The activity of geranylgeranyl reductase is more affected by low temperature (273 K) than that of chlorophyll synthase (Schoefs and Bertrand, 2000). Cycloheximide is an inhibitor of the hydrogenation of geranylgeranyl to phytyl moiety in the irradiated etiolated seedlings, whereas it has no effect on Chlide esterification with geranylgeranyol (Rassadina et al., 2004).

### Chlorophyll *b* Formation

Chl *b* is formed from Chlide in two reactions (Figure 1). The first is the two-step oxygenation of the side methyl group at the C7 in the ring B to a formyl group using molecular oxygen, which is catalyzed by chlorophyllide a oxygenase (CAO; EC 1.14.13.122) (Tanaka et al., 1998; for review see Rüdiger, 2002, 2006; Tanaka and Tanaka, 2007). Chlide *a* was shown to be the sole substrate for CAO activity, because neither Chl *a* nor Pchlide were accepted as the substrates of CAO *in vitro* (Oster et al., 2000; Rüdiger, 2002). The CAO is a membrane-bound protein, localized in thylakoid and envelope membranes (Eggink et al., 2004). The biosynthesis of Chl *b* plays an important regulatory role in the assembly and stabilization of light harvesting antennae (LHC) and as a consequence in granum stacking, which rely on the presence of this pigment (Espineda et al., 1999; Tanaka et al., 2001; Harper et al., 2004; Reinbothe et al., 2006). Judging from the low activity of the recombinant CAO *in vitro* it was suggested that additional accessory proteins might be required to reach the optimal catalytic oxygenase activity (Eggink et al., 2004), but these are yet unidentified.

Esterification of the D ring of Chlide, a reaction which is described in section Esterification of Chlide for Chl *a* formation, converts Chlide *b* into Chl *b*. The Chl *a/b* ratio in plants is regulated using the reversible conversion of Chlide *a*—Chl *b*—Chl(ide) *a*, which is known as “the chlorophyll cycle” (for a review see Rüdiger, 2002; Tanaka and Tanaka, 2019). Keeping the optimal level of Chl *a/b* ratio which correlates with the amount of the LHC complexes and supramolecular structure of photosynthetic complexes enables plant adaptation to changing environmental conditions (for a review see Tanaka and Tanaka, 2011; Voitsekhovskaja and Tyutereva, 2015).

### Late Chlorophyll Biosynthesis Is Associated With Plastid Membranes

The Mg-branch occurs at the plastid membranes and some enzymes are organized into macromolecular complexes. For example, LIL3, a protein belonging to LHC family, plays an essential role in the organization of complexes involved in Chl biosynthesis and in the delivery of Chls to photosynthetic complexes. By linking tetrapyrrole and terpenoid biosynthesis, LIL3 plays a critical role in the organization of later steps in Chl biosynthesis (Hey et al., 2017). Studies on the Arabidopsis mutant lacking LIL3 revealed the importance of this protein for Chl biosynthesis and in the stabilization of geranylgeranyl reductase (Tanaka et al., 2010). A direct interaction of geranylgeranyl reductase with LIL3 was demonstrated using a split ubiquitin assay, bimolecular fluorescence complementation as well as combined blue-native and SDS polyacrylamide gel electrophoresis (Tanaka et al., 2010). LIL3 was also shown to stabilize LPOR, and the direct LIL3-LPOR interaction was also confirmed using multiple analysis of protein-protein interactions (Hey et al., 2017). In addition to that, fluorescence *in vitro* analysis showed high binding affinity of LIL3 to Pchlide—the substrate of LPOR. However, no interactions with chlorophyll synthase were reported in this study. Similar complexes were detected in etio-chloroplasts and etioplasts of barley, using native gel electrophoresis with autofluorescence detection and mass spectrometry (Reisinger et al., 2008; Mork-Jansson et al., 2015). In these experiments, LIL3 formed complexes with LPOR, geranylgeranyl reductase and chlorophyll synthase. However, using split ubiquitin assay, the interaction between LIL3, LPOR and chlorophyll synthase was demonstrated, whereas no interaction with gerenylgeranyl reductase was proven (Mork-Jansson et al., 2015). Studies performed on thylakoids of rice (Zhou et al., 2017) revealed an additional protein called geranylgeranyl reductase recruiting protein, regulating the binding of geranylgeranyl reductase in the complexes clustered around LIL3.

Membrane complexes composed of Mg-protoporphyrin IX monomethylester cyclase, CHL27, LPOR (i.e., LPOR-B, LPOR-C), geranylgeranyl reductase and the FLU protein were identified in isolated thylakoids of Arabidopsis (Kauss et al., 2012). FLU is a negative regulator of Chl biosynthesis operating at the step of delta aminolevulinic acid (ALA) formation (Meskauskiene et al., 2001; Meskauskiene and Apel, 2002). In the absence of light, glutamyl-tRNA reductase is bound to FLU in these complexes and ALA formation is inhibited (Zhang et al., 2015). Formation of complexes of FLU with enzymes catalyzing the final steps of Chl biosynthesis was demonstrated using native electrophoresis, immunoprecipitation and mass spectrometry (Kauss et al., 2012).

It was also shown that, Mg-protoporphyrin IX monomethylester cyclase forms complexes with YCF54 and FNR1. However, YCF54 is probably a scaffold protein for a multi-subunit enzymatic complex, including other enzymes of late Chl biosynthesis, namely LPOR as well as the DVR and geranylgeranyl reductases (Kong et al., 2016; Herbst et al., 2018). Formation of such multi-subunit complexes favors the flow of intermediates during the Chl synthesis in light as well as the inhibition of the process in the dark. Taking into account that FNR1 provides the electrons for the cyclase activity, it is hypothesized that it might also deliver electrons for LPOR and DVR in the multi-enzymatic complex (Herbst et al., 2018, 2019). Nevertheless, the direct interaction of YCF54-FNR1 with LPOR and DVR has not yet been shown until now. Moreover, the question about the regulation of the electron flow in thylakoids remains open. Finally, it has not been elucidated how the late Chl synthesis is orchestrated at the beginning of angiosperm greening.

Another question which also remains open till date concerns the coexistence of CHL27-YCF54-FLU-LPOR-geranylgeranyl reductase complex with the LIL3-LPOR-geranylgeranyl reductase-geranylgeranyl diphosphate synthase-chlorophyll synthase complex in thylakoid membranes. LPOR and geranylgeranyl reductase were found in both type of complexes in thylakoid membranes. However, it remains unknown whether these complexes are somehow associated or stay separated. Recent analyses revealed that in Arabidopsis LPOR, curvature Thy1 (CURT1), the Mg^2+^-chelatase subunit 1 (CHLI) and Mg^2+^ protoporphyrin IX methyl transferase (CHLM) are also located to the granum margins (Wang et al., 2020). Further research is required to elucidate the exact localization, organization and regulation of the last steps of Chl biosynthesis. Answering these questions is important for the understanding of the regulatory mechanisms of the delivery of prenyl intermediates for Chl and tocopherol synthesis (Gutbrod et al., 2019).

### Dual Localization of the Mg-branch in Plastids and the Role of Lipids in It

Dual localization of enzymes involved in the Mg-branch in both chloroplast envelope and plastid inner membranes were documented based on biochemical studies (Joyard et al., 1990; Block et al., 2002; Tottey et al., 2003; Eggink et al., 2004; Tanaka and Tanaka, 2007), and later confirmed by proteomics (for review see Joyard et al., 2009; Bruley et al., 2012; Salvi et al., 2018). Dual localization was suggested to supply Chl to different Chl-binding proteins (PS core complexes and LHC) (Tottey et al., 2003). Additional research is required to elucidate the functional meaning of the dual localization and its relation to protein import mechanisms of plastids. Some yet unsolved problems that need to be mentioned: (i) interactions among enzymes and regulatory factors, (ii) structure and functioning of enzyme complexes, and (iii) their interaction with lipid membrane components, as well as (iv) the MV and DV heterogeneity of the biosynthesis route.

An interesting research area is the understanding of the regulatory networks connecting the biosynthesis of Chls, of protein components of photosynthetic complexes as well as lipids with the formation of the thylakoid membranes typical for chloroplasts. These processes need to be orchestrated to enable the proper assembly of the photosynthetic apparatus and to avoid the overaccumulation of unbound tetrapyrroles which may lead among others to photooxidative damage (Erdei et al., 2005; Hideg et al., 2010; Kim et al., 2013). It is nowadays known that the expression of Mg-chelatase and Mg-protoporphyrin IX monomethyl ester cyclase is linked to the synthesis of galactolipids (Fujii et al., 2014; Kobayashi et al., 2014; Kobayashi, 2016, 2018; Kobayashi and Masuda, 2016). Coordination of Chl and galactolipid biosyntheses goes through cytokinin and light signaling pathways (Kobayashi et al., 2014), however, further studies are needed to understand the molecular background of this regulation.

It has been shown that in etioplasts, the Mg-branch is sensitive to the membrane lipid environment, namely the MGDG and DGDG levels. Deficiency of any of these galactolipids in *A. thaliana* mutants resulted in overaccumulation of Mg-protoporphyrin IX pointing to the impairment of the following enzymes in the biosynthetic pathway (Figure 1) and leading to the accumulation of Pchlide intermediates and low Pchlide content (Fujii et al., 2017, 2018). On the contrary, the Mg-branch was reconstituted in *E. coli* cells from recombined *Synechocystis* proteins: Mg-chelatase, Mg-protoporphyrin IX methyltransferase, Mg-protoporphyrin IX monomethyl ester cyclase, LPOR, DVR, Chl synthase, and geranylgeranyl reductase without galactolipids (Chen et al., 2018). Thus, galactolipids rather enhance the activity of the enzymes, however, the regulatory mechanisms are yet unknown. Functioning of Mg-protoporphyrin IX methyltransferase, Mg-protoporphyrin IX monomethyl ester cyclase in barley etioplasts was also affected in the absence of carotenoids (La Rocca et al., 2007).

## LPOR—An Enzyme Operating in Lipid Environment

Among all reaction steps of Chl biosynthesis, the photoreduction of Pchlide is probably the most studied. This may be a result of several factors including (i) the unique catalytic activity of the enzyme involving an ultrafast light-activated photochemical reaction interesting from the biophysical and biochemical points of view, (ii) its important role in the regulation of tetrapyrrole biosynthesis, (iii) its association with arrested chloroplast differentiation and the formation of peculiar etioplast inner membranes, and (iv) the fact that dark-grown angiosperm seedlings represent a convenient plant material to study the last steps of the biosynthetic pathway in a synchronized way after illumination. Spectroscopy is also a useful tool to study this topic because there is a 30–40 nm difference in the spectral properties of the substrate, Pchlide (a porphyrin containing 11 double bonds in the tetrapyrrole ring) and the product, Chlide (a chlorine with 10 double bonds). This way and due to the high sensitivity of the delocalized electron system of the porphyrin ring to alterations in the molecular environment of the pigment, the reaction as well as the different populations of the pigments can be easily studied and characterized by relatively simple steady-state spectroscopic methods, which is summarized in the section Spectroscopic Properties of Pchlide *in vivo*. Pigments involved in similar molecular interactions and located in similar molecular micro-environments within the plastids exhibit similar spectral properties and thus represent a subpopulation of the entire pigment pool denoted as a pigment “form.” Pigment forms are often referred to by using the wavelength of their spectral maxima, but most of them have been also characterized from the biochemical and physiological points of view.

However, it is important to mention, that in green leaves containing significant amounts of Chls in the form of the various Chl-protein complexes of the photosynthetic apparatus, the fluorescence emission signal of Chl hinders the detection of its precursors such as e.g., Pchlide, which are present in much lower quantities (i.e., three order of magnitude lower amounts). Similarly, in green plants, the level of enzymes required for the steady-state Chl synthesis that produces Chl molecules to replace the pigments damaged during photosynthesis or stress conditions is very low and is hard to detect with classical biochemical or spectroscopic methods. Therefore, most studies related to the last steps of Chl biosynthesis have been performed on dark-germinated angiosperm seedlings in which the light-dependent LPOR enzyme is accumulating along with its substrate Pchlide, while Chl is absent.

When deprived from light during the early stages of their development, proplastids differentiate into a peculiar plastid type called etioplast in the photosynthetic tissues of dark-grown plants. The inner membranes of etioplasts consist of flat, sac-like membranes called prothylakoids, i.e., lipid bilayers encircling an inner aqueous phase called lumen, and a unique non-lamellar, but cubic phase inner membrane structure, the prolamellar body (PLB). Due to the inhibition of other light-signaling pathways [e.g., those involving phytochrome and cryptochrome (Sineshchekov and Belyaeva, 2019)] in darkness, the development of such dark-grown seedlings is also peculiar and is called skotomorphogenesis. When etiolated plants get illuminated, photomorphogenesis proceeds in them in parallel with the light-induced conversion of Pchlide to Chlide, resulting in the etioplast-to-chloroplast conversion which includes major reorganization of the plastid inner membranes, and active synthesis of the components of the photosynthetic apparatus (reviewed in Solymosi and Aronsson, 2013; Armarego-Marriott et al., 2020; Hernández-Verdeja et al., 2020).

Data about LPOR and its native structure and activity are reviewed below with emphasis on the lipid-protein interactions in them.

### Biochemical Characterization of LPOR

LPOR belongs to the short-chain dehydrogenase/reductase (SDR) protein superfamily (Yang and Cheng, 2004). It is a nuclear encoded protein which is then post-translationally imported to plastids (Aronsson et al., 2003b; Kim et al., 2005). Based on its amino acid sequence it is considered as a soluble and globular protein with surprisingly high contents of basic and hydrophobic amino acids (Schoefs and Franck, 2003; Masuda and Takamiya, 2004; Heyes and Hunter, 2005; Gabruk and Mysliwa-Kurdziel, 2015). Circular dichroism studies and bioinformatic tools predicted its secondary and tertiary structure (Schulz et al., 1989; Darrah et al., 1990; Birve et al., 1996; Dahlin et al., 1999; Townley et al., 2001; Buhr et al., 2008; Gabruk et al., 2012, 2015; Menon et al., 2016; Gholami et al., 2018), but hydropathy plots did not identify potential hydrophobic transmembrane segments in it (Benli et al., 1991; Spano et al., 1992). This is surprising as 98% of LPOR present in etioplasts has been localized to PLB membranes (Ryberg and Sundqvist, 1982a; Ikeuchi and Murakami, 1983), and it represents the major protein of isolated PLB fractions (Ryberg and Sundqvist, 1982a; Selstam and Sandelius, 1984; von Zychlinski et al., 2005; Blomqvist et al., 2008; Kanervo et al., 2008). Several *in vitro* and *in vivo* experiments involving washing and directed mutagenesis tried to identify the membrane association mechanism of LPOR. It has been shown that NADPH and ATP are required for its proper binding to plastid inner membranes such as PLBs, PTs and thylakoids (Dahlin et al., 1995; Engdahl et al., 2001). Washing experiments with various salts, detergents (Grevby et al., 1989; Selstam and Widell-Wigge, 1989) and proteases (Lütz and Tönissen, 1984; Dahlin et al., 1995; Engdahl et al., 2001) also revealed that LPOR binds more strongly to etioplast inner membranes (PLBs and PTs) than to thylakoid membranes. In chloroplasts, LPOR was predominantly found in the granum margins (Wang et al., 2020) which are, similarly to PLBs, also highly curved membranes with special lipid composition and special membrane organization. Based on its amino acid sequence LPOR is not an integral transmembrane protein, but because of its strong attachment to PLBs and PTs due to which it is difficult to solubilize it, it is probably not a peripheral membrane protein but an integral monotopic membrane protein which is permanently attached to one side of the plastid inner membrane. Using mutagenesis experiments, some amino acid residues (Cys) and the C-terminal have been shown to be involved in membrane association of LPOR (Dahlin et al., 1999; Aronsson et al., 2001), and recently a Chaperone-like Protein of POR1 (CPP1) has been identified which may be involved in anchoring LPOR to the PLBs (Lee et al., 2013b).

LPOR has a central β-sheet comprised of β-strands surrounded by α-helices, forming a typical dinucleotide binding Rossmann fold (Rossmann et al., 1974). The catalytic YxxxK and the NADPH-bounding G-rich (GxxxGxG) motifs are conservative (Wilks and Timko, 1995; Schoefs and Franck, 2003; Gabruk and Mysliwa-Kurdziel, 2020). LPOR crystal structure remained unknown for a long time; the structures of two cyanobacterial LPOR enzymes have been published only recently (Zhang et al., 2019; Dong et al., 2020). In general, both structures are compatible with the homology models, however, due to slightly different protocols there were some differences between these two works.

Most SDR proteins have tendency to form dimers and oligomers (Jörnvall et al., 1995), a property which was also found in LPOR macrocomplexes isolated from PLBs pre-treated with chemical cross-linkers (Wiktorsson et al., 1993), or isolated in fully photoactive native state from PLBs after mild solubilization and gel chromatography (Ouazzani Chahdi et al., 1998). Circular dichroism spectra (Mathis and Sauer, 1972; Böddi et al., 1989, 1990) and energy transfer studies (Kahn et al., 1970) also revealed that dimers or oligomers of the pigments are involved in the photoreduction of Pchlide. Cross-linking and subsequent mass spectrometric analysis of recombinant LPOR suggested that after substrate binding, structural changes occur in the LPOR oligomers which bring the catalytic motifs and the Pchlide molecules bound to the active site closer together (Gabruk et al., 2016). Similar observations were done in case of the analyses of cyanobacterial LPOR in which substrate binding induced oligomerization (Zhang et al., 2019) or monomers were observed in solution but homodimerization was observed during crystal formation (Dong et al., 2020). Experiments with recombinant pea LPOR also showed that it can form photoactive dimers in solution (Martin et al., 1997). A close distance between catalytic motifs brings the Pchlide molecules bound within the oligomers into close proximity, which enables energy transfer between them and also influences their spectral properties (Kahn et al., 1970). It has to be noted that the enzymatically active LPOR complexes have unique spectral and biochemical properties that are hardly reconstituted *in vitro*, especially in the absence of lipids (Gabruk et al., 2017). However, recent *in vitro* reconstitution studies successfully yielded crystal structures of oligomers: in case of cyanobacterial LPOR octamers were reported (Zhang et al., 2021) while in case of Arabidopsis LPOR helical structures associated with lipids were observed (Nguyen et al., 2021).

Data indicate the role of carotenoids [zeaxanthin and violaxanthin (Ouazzani Chahdi et al., 1998); neoxanthin and violaxanthin (Bykowski et al., 2020); the accumulation of poly-cis xanthophylls (Park et al., 2002; Cuttriss et al., 2007)] and lipids [MGDG (Aronsson et al., 2008; Fujii et al., 2017); and MGDG, PG, and SQDG (Gabruk et al., 2017; Nguyen et al., 2021)] in the formation of the photoactive enzyme complexes and the PLBs. Similarly, carotenoids (Denev et al., 2005) were suggested to be involved in the membrane association of LPOR. Lipid biosynthesis mutants had hindered LPOR activity, no LPOR oligomerization and abnormal PLB formation (Fujii et al., 2017, 2018, 2019b) which again outlines the strong relation between the LPOR oligomers, plastid lipids and inner membrane structures. Cryo electron microscopic investigations on *in vitro* reconstituted LPOR revealed that LPOR oligomers form helical filaments around lipid bilayer tubes and are inserted in the outer leaflet of the membranes (Nguyen et al., 2021).

**Figure 2:**
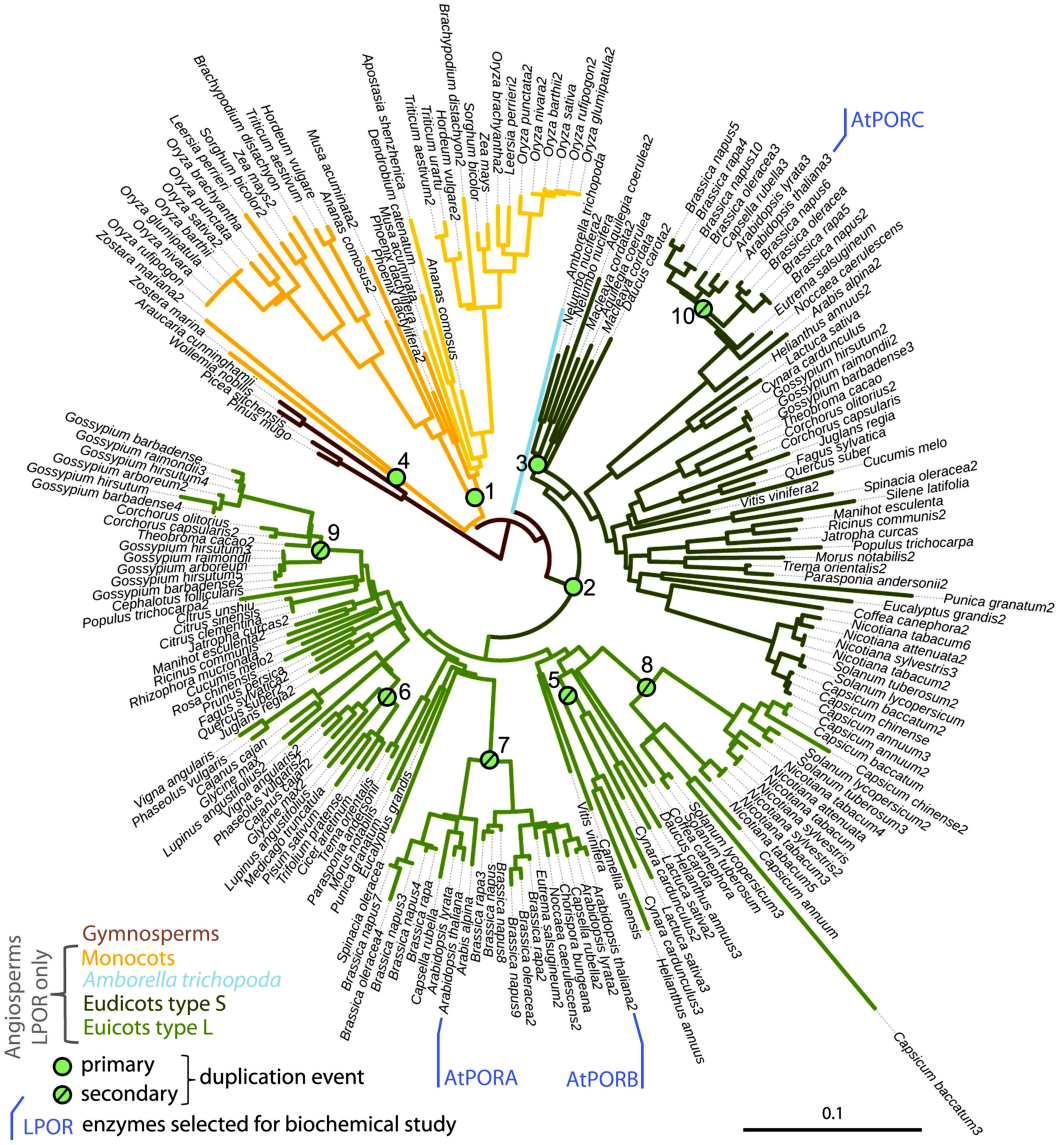
Subtree of LPOR sequences from seed plants, with *Arabidopsis thaliana*'s isoforms AtPORA, AtPORB, and AtPORC being marked, and green dots indicating duplication events. Republished with permission of Portland Press Ltd., from The origin, evolution and diversification of multiple isoforms of light-dependent protochlorophyllide oxidoreductase (LPOR): Focus on angiosperms; Gabruk and Mysliwa-Kurdziel (2020), permission conveyed through Copyright Clearance Center, Inc.

*LPOR* gene probably appeared ~1.36 billion years ago, and since then it underwent several duplication events and mutations (Figure 2) (Gabruk and Mysliwa-Kurdziel, 2020). As a result, few organisms contain only one LPOR gene and thus one isoform [e.g., pea (Spano et al., 1992) and cucumber (Fusada et al., 2000)], but several organisms contain at least two different isoforms (termed in general or historically as LPOR-A and LPOR-B—barley, rice, tobacco, and wheat, see later for references) or even more than that [e.g., Arabidopsis also has LPOR-C (Oosawa et al., 2000; reviewed in Gabruk and Mysliwa-Kurdziel, 2020)]. The isoforms share ~75% sequence homology, but according to detailed functional analyses performed on a few species—barley (Holtorf et al., 1995; Garrone et al., 2015), Arabidopsis (Armstrong et al., 1995), tobacco (Masuda et al., 2002), wheat (Blomqvist et al., 2008), and rice (Kwon et al., 2017)—they have different expression patterns, substrate binding affinities, different plastid import mechanisms, different localization within the plastid membranes and different gene regulation patterns also depending on the developmental stage, temperature and light conditions (reviewed in Solymosi and Schoefs, 2008, 2010; Gabruk and Mysliwa-Kurdziel, 2020). The transport of the different isoforms into the plastids is peculiar as it shows a large substrate, cell, tissue, and organ specificity (Aronsson et al., 2003b; Kim et al., 2005), the detailed discussion of which is beyond the scope of this review.

LPOR-A and LPOR-B are present in etiolated material, while LPOR-C is expressed typically in green tissues (Oosawa et al., 2000; Aronsson et al., 2003a; Masuda et al., 2003; reviewed in Solymosi and Schoefs, 2010; Gabruk and Mysliwa-Kurdziel, 2015). LPOR-A is transiently expressed during early phases of development when large amounts of pigments need to be synthesized quickly, while LPOR-B and LPOR-C are thought to be responsible for the bulk Chl synthesis of adult or green plants (Paddock et al., 2010, 2012). Arabidopsis double mutants lacking LPOR-B and -C were shown to be unable to produce enough Chl under light conditions, indicating the importance of these isoforms in the biogenesis of the photosynthetic apparatus and also its membrane structures such as grana (Frick et al., 2003). Some ancestral LPOR genes (like that of *Synechocystis*) may even have different catalytic activity as they operate in lipid independent manner in contrast with other LPORs analyzed so far in angiosperms or gymnosperms (Gabruk and Mysliwa-Kurdziel, 2020). Taking into account the biochemical characteristics of LPOR isoforms, their interaction with lipids, as well as their phylogenetic relationships, Gabruk and Mysliwa-Kurdziel (Gabruk and Mysliwa-Kurdziel, 2020) proposed a new classification of LPOR family consisting of three LPOR types (Figure 2). The first one includes bacterial LPORs, termed “Z-type,” which are lipid-independent. Two other categories, termed L-type and S-type LPOR isoforms, are lipid-driven and present in angiosperms. L-type isoforms preferentially form complexes on the lipid membranes (like *A. thaliana* LPOR-A and LPOR-B), while the S-type ones (like *A. thaliana* LPOR-C) are active both with and without lipids. Further biochemical investigation is required to characterize the effect of lipids on LPOR isoforms from other angiosperm species.

### Spectroscopic Properties of Pchlide *in vivo*

**Figure 3:**
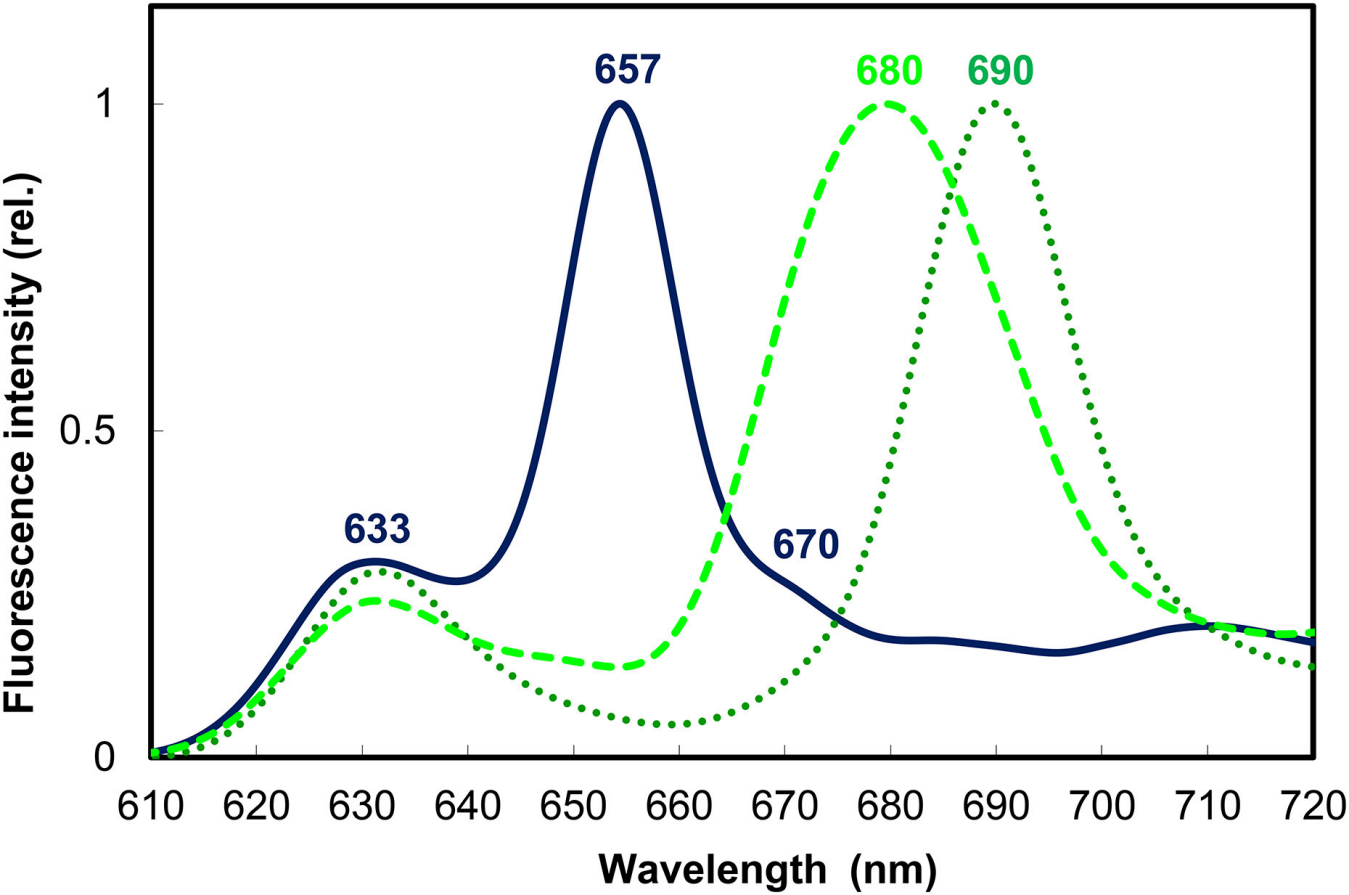
Normalized 77 K fluorescence emission spectra of 10-day-old dark-grown wheat (*Triticum aestivum*) leaves before (solid line) and after illumination with white light of 100 μmol s^−1^ m^−2^ photon flux density for 10 s (dotted line) and a subsequent 15 min dark incubation to reach the end stage of Shibata shift (broken line). Experimental conditions were as in Solymosi et al. (2002) and Smeller et al. (2003).

Native spectral properties of Pchlide complexes in etiolated seedlings, and the spectral changes following the light-induced reduction of photoactive Pchlide were described already in the 50's (Figure 3) (Shibata, 1957). Photoreduction of Pchlide can take place at 203 K and reversible intermediates can be observed already at 77 K (Sironval and Brouers, 1970; Heyes et al., 2002, 2003; Belyaeva and Litvin, 2011, 2014), therefore, low temperature (typically 77 K) spectroscopic methods are needed to characterize the native state of the pigments. The absorption and fluorescence emission spectra of etiolated leaves contain two major spectral bands, each attributed to a specific pigment form also characterized at the biochemical level. Since low temperature absorption spectroscopy is less frequently used, we will refer to 77 K fluorescence properties of the pigments in this work.

The short-wavelength band with fluorescence emission maximum at 633 nm represents a pool of monomeric Pchlide pigments bound either to the membrane surface or to a yet unidentified protein or to LPOR, but not in the active site of the enzyme or not to the active form of the enzyme (Figure 3). These pigments were primarily localized to the prothylakoid membranes of the etioplasts (Ryberg and Sundqvist, 1982a,b) and to the cytosolic side of the outer envelope (Joyard et al., 1990). They are not directly photoconvertible with a flash, and thus belong to the so-called “non-photoactive” Pchlide pool.

Another major band located at 655–657 nm belongs to the so-called “photoactive” Pchlide pool, i.e., Pchlide pigments bound to the active site of LPOR macrodomains, which correspond to oligomers of Pchlide:LPOR:NADPH ternary complexes and are strongly associated with the PLB membranes of the etioplasts (Figure 3) (Ryberg and Sundqvist, 1982a,b). Gaussian deconvolution and further spectral and biochemical analyses of isolated and fractionated etioplast inner membranes revealed the presence of other minor Pchlide forms, like for instance smaller oligomers (probably dimers) of Pchlide:LPOR:NADPH ternary complexes with emission maximum at 644 nm which are also photoactive and are suggested to be located to the edge of the PLB membranes (Böddi et al., 1990, 1991, 1992). In addition, a non-photoactive Pchlide molecular subpopulation hypothetically located to the central regions of PLBs and having fluorescence emission maximum at around 670 nm was also described (Figure 3) (Böddi et al., 1990; Bykowski et al., 2020). Upon short (already μs-long) illumination the fluorescence emission of Pchlide:LPOR:NADPH oligomers with emission maxima at 655 nm disappears, and that of the freshly produced Chlide:LPOR:NADP^+^ oligomers appears at 690 nm (Figure 3) (Böddi et al., 1990). After 10–15 min these oligomers and the pigments located in them undergo conformational changes and disaggregation as reflected by the blue shift of their emission maximum toward 680 nm, referred to as the so-called Shibata shift (Figure 3) (Shibata, 1957; Smeller et al., 2003; Solymosi et al., 2007a).

After the ultrafast transformation of the LPOR-bound, so-called “photoactive” Pchlide molecules into Chlide, and the subsequent slower conformational changes and reorganizations of LPOR oligomers including the dissociation of Chlide, the so-called “non-photoactive” Pchlide molecules can bind to the active site of LPOR and can be then also directly transformed into Chlide in the same ultrafast photochemical reaction step.

Depending on the oxidation state of NADPH in the ternary complexes, and also on the studied species and organs (e.g., leaves or stems) other spectral forms have been also characterized but discussion about them, as well as about developmental and other species-specific factors determining the ratios of the different forms is beyond the scope of this review, and can be found e.g., in Schoefs (2005), Belyaeva and Litvin (2007), and Solymosi and Schoefs (2008, 2010). Below we summarize knowledge about the molecular background of Pchlide photoreduction and the membrane association of LPOR.

### Understanding the Photophysical and Spectral Properties and Molecular Organization of Pchlide and LPOR

The major hurdle in understanding LPOR structure and its exact molecular interaction with PLB membranes is the fact that the *in vivo* crystal structure of photoactive LPOR complexes is not available so far. In addition, photoactive dimeric and oligomeric complexes of LPOR are also hard to be isolated and fully purified from etiolated tissues because they often undergo disaggregation and dissociation during these processes. As stated earlier, it is even harder to detect or isolate them from fully green plant material. On the other hand, photoactive LPOR is also hard to reconstitute *in vitro*. Below we'll review some key spectral and structural features of Pchlide and LPOR, and also discuss how various *in vitro* experiments and reconstitution studies helped us to better understand the native structure of LPOR.

#### Photophysical and Spectral Properties of Pchlide in Solvents and Lipid Model Systems

The key role of Pchlide in the light-triggered biosynthesis of Chl is related to its electronic properties. The Pchlide molecule captures sunlight and uses the absorbed energy to power the enzymatic reduction of its C17–C18 double bond. Data for understanding Pchlide photochemistry were collected from numerous studies of Pchlide in various model systems. It is now well-documented that Pchlide is an intrinsically reactive molecule. After light absorption, an electronically excited Pchlide molecule undergoes deexcitation using two parallel pathways (Dietzek et al., 2004, 2006b, 2009; Colindres-Rojas et al., 2011). The first one, called the reactive pathway, goes through an intramolecular charge-transfer state (S_ICT_), which is non-fluorescent. The other, called the non-reactive pathway, goes through the lowest excited singlet state (S_1_), which then decays *via* fluorescence or by intersystem crossing to the long-lived Pchlide triplet state. Charge separation across Pchlide molecule in the S_ICT_ state depends on solvent polarity and is more stable in polar solvents (Dietzek et al., 2006a, 2010). A carbonyl group (C13) in the Pchlide isopentanone ring is of special importance for this charge separation (Sytina et al., 2010; Heyes et al., 2017), and the formation of the S_ICT_ state is important for Pchlide photocatalysis (Dietzek et al., 2006b, 2010; Heyes et al., 2015).

Fluorescence emission spectra of Pchlide in organic solvents have the maximum between 626 and 641.5 nm (Mysliwa-Kurdziel et al., 2004). The photophysical properties of Pchlide S_1_ state only weakly depend on non-specific solvation, as revealed from the small Stokes shifts (between 50 and 300 cm^−1^) observed in organic solvents (Mysliwa-Kurdziel et al., 2004). Specific solvation in protic (methanol, ethanol) and coordinating solvents (pyridine) enlarges the Stokes shift due to the lowering of the S_1_ state energy, and shortens the fluorescence lifetime. The site-specific solvation in Pchlide excited state *via* hydrogen bonding was confirmed experimentally (Mysliwa-Kurdziel et al., 2004; Sytina et al., 2010) and by theoretical calculation (Zhao and Han, 2008). DV-Pchlide has slightly red-shifted absorption and fluorescence maxima and differs in fluorescence lifetime when compared to MV-Pchlide (Kotzabasis et al., 1990; Kruk and Mysliwa-Kurdziel, 2004; Mysliwa-Kurdziel et al., 2008). The prenyl moiety, present in protochlorophyll molecule, only slightly changes the spectral and photophysical properties of the tetrapyrrole ring of Pchlide (Mysliwa-Kurdziel et al., 2004, 2008).

Pchlide in aqueous solutions forms aggregates even at low concentrations, which is manifested by a significant red shift of the absorption and emission maxima, and in strong fluorescence quenching (Mysliwa-Kurdziel et al., 2004, 2013b; Sytina et al., 2011a,b). Aggregation of the pigments was also observed in organic solvents at high Pchlide concentrations (Kotzabasis et al., 1990; Kruk and Mysliwa-Kurdziel, 2004; Mysliwa-Kurdziel et al., 2013b).

Liposomes composed of thylakoid lipids were used to model interactions of free Pchlide molecules with etioplast inner membranes (Mysliwa-Kurdziel et al., 2013a,b). Pchlide molecules were found at the interface area of the liposomes and/or the head-group area of the lipid bilayer. In the case of high Pchlide contents, aggregate formation was observed, which was facilitated in galactolipid liposomes. Pchlide aggregates had similar fluorescence emission to aggregated Pchlide:LPOR:NADPH complexes *in vivo* (i.e., at 656 nm), however, the excitation maximum was red-shifted to 480 nm. Experiments performed for Pchlide in reversed micelles from dioctyl sulfosuccinate sodium salt (AOT)/isooctane mixture showed that the molecular dynamics of water bound at the hydrophylic core of micelles is also important for Pchlide monomer-aggregate equilibrium (Mysliwa-Kurdziel et al., 2013b).

**Figure 4:**
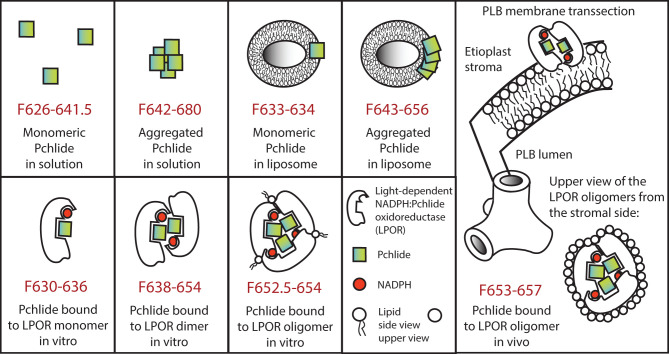
Model representing spectral properties and molecular interactions of protochlorophyllide in various *in vitro* model systems as well as *in vivo* in case of the major photoactive protochlorophyllide form located to the prolamellar bodies. In case of oligomers for simplicity trimers are represented, although larger aggregates (e.g., tetramers and octamers) have been also reported. For further details see the text.

As observed in the experiments performed with *in vitro* enzyme-free model systems, the Pchlide chromophore is very sensitive to changes in its molecular environment. In the monomeric state its fluorescence emission varies between 626 and 641.5 nm, fluorescence emission maxima of Pchlide pigment forms above 641.5 nm can only be obtained when two or more Pchlide molecules are in close proximity, have overlapping delocalized electron systems and thus form aggregates *in vitro* (see above, and Mysliwa-Kurdziel et al., 2004, 2013a,b; Sytina et al., 2011a,b). A model summarizing the spectral properties and the molecular interactions of Pchlide *in vitro* is presented in Figure 4.

#### Spectral Properties of *in vitro* Reconstituted Pchlide:LPOR:NADPH Complexes

Based on data obtained from *in vitro* model systems as well as other experimental evidence including CD spectroscopy of etioplast inner membrane fractions isolated from etiolated leaves (Böddi et al., 1989, 1990), it seems clear that the photoactive Pchlide complexes contain Pchlide pigments in at least dimeric or aggregated forms. This can be probably achieved by the distinct conformation of the LPOR dimers or oligomers in which the delocalized electron systems of neighboring Pchlide molecules overlap. Below we'll briefly review the latest *in vitro* reconstitution experiments of photoactive LPOR complexes. Again, we should mention that important pioneering observations have been done on isolated and partially purified photoactive complexes (in early works referred to as holochromes) from plants, but detailed discussion of these complexes and early data is provided elsewhere (e.g., Schoefs and Franck, 2003; Solymosi and Schoefs, 2010). Similarly, we only discuss data on *in vitro* reconstitution experiments of photoactive plant LPOR complexes, and not those about cyanobacterial LPOR (reviewed e.g., in Heyes and Hunter, 2005), because the peculiar membrane structures (prolamellar bodies) and direct LPOR-lipid and LPOR-membrane interactions have been only scarcely studied in such organisms (Schneidewind et al., 2019; Yamamoto et al., 2020).

Martin et al. (1997) successfully reconstituted photoactive complexes from recombinant pea LPOR with absorption maximum at around 630 nm, which were—based on their molecular mass—assigned to LPOR dimers. However, plant LPOR complexes with fluorescence emission maximum at 655 nm were reconstituted for the first time using a mixture of LPOR isoforms from barley (i.e., LPOR-A and LPOR-B), two different zinc derivatives of Pchlide as well as lipids extracted from isolated PLBs (Reinbothe et al., 1999). Later *in vitro* experiments using Pchlide and recombinant LPOR-A from *Arabidopsis thaliana* demonstrated that the formation of ternary LPOR complexes with fluorescence emission maximum at 655 nm requires the presence of MGDG and a negatively charged plant lipid (either PG or SQDG) (Figure 4) (Gabruk et al., 2017; Nguyen et al., 2021). Lipids did not only play a kind of structural role but also influenced the enzyme activity. Negatively charged lipids (PG and SQDG) did not influence the spectral properties of the complexes but affected their NADPH binding properties. When only PG was present, much lower concentrations of NADPH were required by the enzyme to form photoactive complexes, suggesting that these LPOR-A complexes are preferably associated to the lipid membranes, especially under low NADPH concentrations which are common in etiolated tissues (Gabruk et al., 2017).

Interestingly, the conical shaped, non-bilayer lipid, MGDG had a strong effect on the spectral properties of LPOR-A complexes *in vitro*. In the presence of MGDG, the fluorescence emission maximum of *in vitro* reconstituted LPOR complexes was shifted up to 652 nm, but the formation of complexes with native-like fluorescence properties (i.e., emission maximum at 655 nm) were only induced in the simultaneous presence of MGDG and PG (Figure 4) (Gabruk et al., 2017). Successful cryo electron microscopic analyses of *in vitro* assembled LPOR, NADPH, Pchlide in a mixture of lipids revealed that LPOR and Pchlide are inserted into the outer leaflet of the membranes, and LPOR forms oligomers arranged in helical filaments which are strongly associated with the membranes and have an important role in inducing and shaping their tubular organization (Nguyen et al., 2021).

### Interaction of Pchlide-LPOR Complexes With Plastid Inner Membranes

As stated above, in etioplasts the photoactive LPOR proteins and their oligomers are mostly located to the PLBs and are only present in minor amounts in isolated prothylakoid fractions, in which monomeric, non-photoactive Pchlide complexes are dominating (Ryberg and Sundqvist, 1982b). LPOR (and especially LPOR-A) accounts for the vast majority of the proteins of the PLBs (Ryberg and Sundqvist, 1982a; Blomqvist et al., 2008).

On the other hand, it has to be added that fluorescence emission typical for monomeric Pchlide has been described in the cytosolic side of the outer envelope membranes of chloroplasts in spinach (Joyard et al., 1990). On the long term (i.e., after 10 min) and in the presence of glycerol slow transformations of the Pchlide pigments was observed in such isolated envelope membrane fractions upon illumination. This indicates that Pchlide-Chlide transformation may take place in the envelope membrane when the LPOR protein conformation is influenced by glycerol (Joyard et al., 1990) which is a component that stabilizes and maybe preferentially favors oligomerization (Zhong et al., 1996; Klement et al., 2000; Solymosi et al., 2002, 2007a; Smeller et al., 2003).

Information about the transport of the LPOR protein from the envelope membranes where they were located (Joyard et al., 1990) toward the internal parts of the plastids is scarce. On the other hand, Dahlin et al. (1995) did not observe accumulation of LPOR in the stroma and the envelope membranes, but have shown that LPOR is a peripheral protein associated with the stromal side of the thylakoid membranes of chloroplasts where it is bound more loosely to the membranes than to PLBs or PTs. Other authors located them to grana margins (Wang et al., 2020). Concerning the import of LPOR from the envelope toward the membranes, it may use plastid vesicle trafficking pathways (Lindquist et al., 2016; Lindquist and Aronsson, 2018).

#### The Unique Membrane Structure of the PLBs

PLBs represent a highly peculiar membrane structure, in which the lipids do not form bilayers, but special cubic phase structures. Such special membranes termed tubular complexes or tubuloreticular inclusions were observed within various intracellular compartments of several organisms including animals, humans or plants. In the former, these are thought to represent the modification of the (rough) endoplasmic reticulum, are in general located inside its cisternae or in the perinuclear space (e.g., Boor et al., 1979) and are rich in acidic glycoproteins. Tubuloreticular complexes have been observed in the cytoplasm of various animal [e.g., dog (Krohn and Sandholm, 1975; Madewell and Munn, 1990), Rhesus monkey (Feldman et al., 1986), cynomolgus monkey (Geisbert et al., 1992), horse and mule (Madewell and Munn, 1989), chicken (Schaff et al., 1976), rat (Datsis, 1993)] and human cells [e.g., lymphoid cells, lymphocytes (Schaff et al., 1972, 1973; Grimley et al., 1973, 1985; Splinter et al., 1975; Kang et al., 1984), monocytes (Luu et al., 1989; Kostianovsky et al., 2016), brain endothelial cells (Lee et al., 1988), liver endothelial cells (Geisbert et al., 1992), heart endothelial cells (Boor et al., 1979), renal cells (Hurd et al., 1969; Grimley et al., 1973; Krohn and Sandholm, 1975; Lee et al., 1988, 2013a, 2017; Datsis, 1993; Elmaghrabi et al., 2017), fibroblasts (Boor et al., 1979; Feldman et al., 1986)] where they were often associated with various, first of all immunological disorders or viral diseases [lupus erythematosus (Hurd et al., 1969; Grimley et al., 1973; Schaff et al., 1973), AIDS (Maturi and Font, 1996), AIDS-associated Kaposi's sarcoma (Marquart, 2005), cytomegalovirus infection (Lee et al., 1988), Sjögren's syndrome (Daniels et al., 1974), SARS (Almsherqi et al., 2005), many other types of virus infections (Deng et al., 2010; reviewed in Grimley and Schaff, 1976; Luu et al., 1989; Almsherqi et al., 2006; Deng et al., 2010) or certain neoplasms [e.g., pituitary tumors (Landolt et al., 1976), connective tissue neoplasms (Madewell and Munn, 1989), plasmacytoma (Madewell and Munn, 1990), intracranial germinomas (Matsumura et al., 1984), lung carcinomas (Schaff et al., 1976), hepatomas (Schaff et al., 1976), Burkitt's type lymphoma (Popoff and Malinin, 1976)].

**Figure 5:**
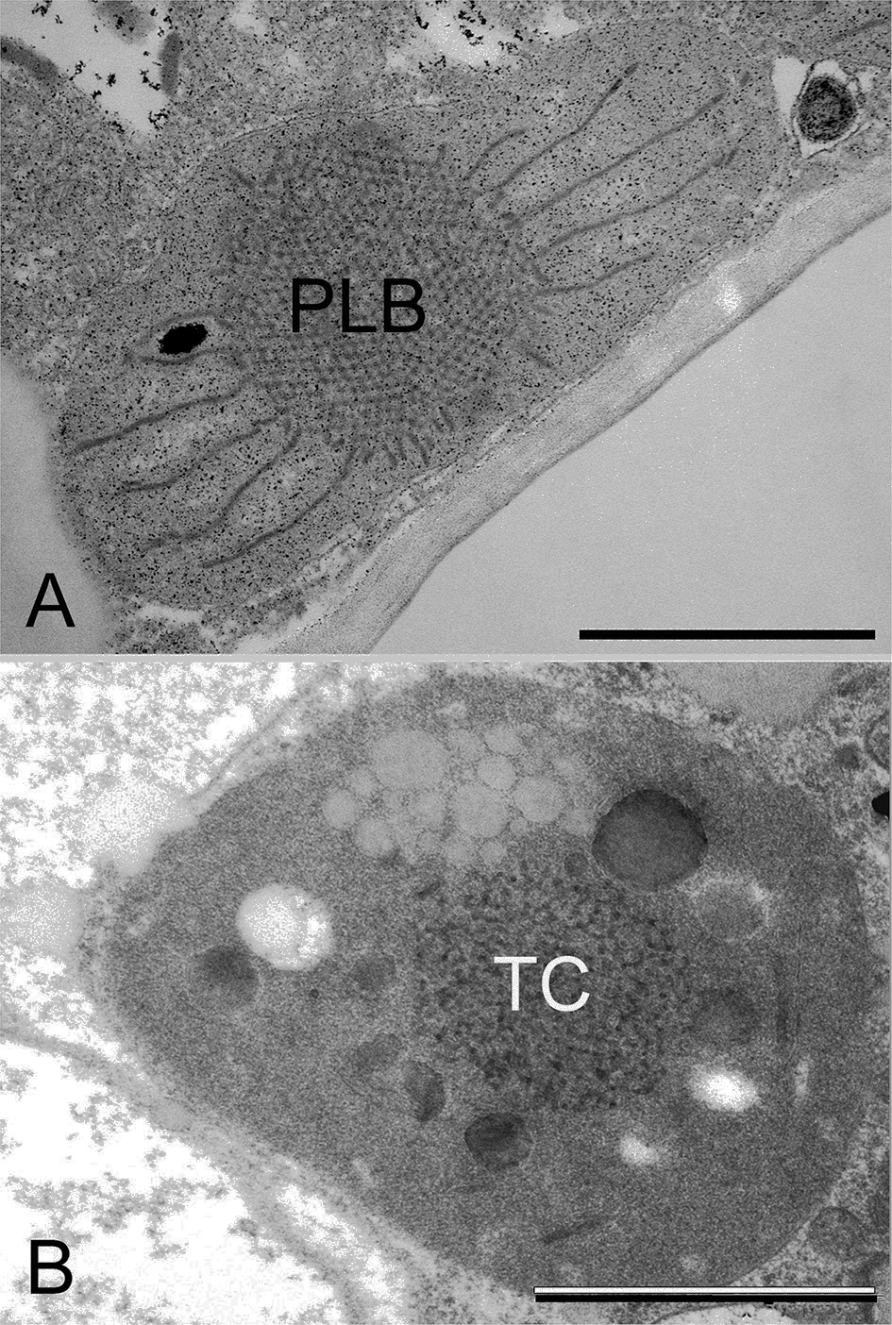
Transmission electron micrographs of the prolamellar body (PLB) present in the etioplast of the cotyledon of a 2-week-old dark-germinated rosemary (*Rosmarinus officinalis*) seedling **(A)**, and tubular complex (TC) of a leucoplast of the neck cell of a peltate glandular hair on the surface of a light-grown adult rosemary plant **(B)**. Scale bar: 1 μm. Sample preparation was as described in Böszörményi et al. (2020).

Tubuloreticular membrane organization has been also observed inside mitochondria and plastids (Almsherqi et al., 2009; Almsherqi, 2010). In the former, cubic membrane formation was induced by starvation and/or autophagy processes of the amoeba cells, during which they were slowing the degradation of the organelle and thus contributed to the survival of the cell upon stress conditions (Chong et al., 2017). In case of plastids, they occurred either in etioplasts or etio-chloroplasts in the form of PLBs and were thus strongly associated with intensive Chl biosynthesis and accumulation of LPOR (Figure 5) (Solymosi and Schoefs, 2008, 2010; Solymosi and Aronsson, 2013) or in secretory plastids having active isoprenoid biosynthesis involving the plastid located MEP-pathway or biosynthesis of hydrophobic molecules such as cutin or suberin (Solymosi and Schoefs, 2010; Böszörményi et al., 2020). Rarely, such tubuloreticular membrane organizations could be interpreted as vesicle clusters (Lindquist et al., 2016) or as structures that appeared under various stress conditions [e.g., UV irradiation (Kovács and Keresztes, 2002)].

Secretory plastids containing tubuloreticular membranes were observed in various taxa and secretory tissues including extrafloral nectaries of *Passiflora* (Schnepf, 1961), plastids of various glandular hairs [e.g., *Cannabis* (Hammond and Mahlberg, 1978; Kim and Mahlberg, 1997; Solymosi and Köfalvi, 2017), *Artemisia* (Ascensao and Pais, 1982), *Chrysanthemum* (Vermeer and Peterson, 1979), *Centrolobium* (Matos and Paiva, 2012), *Platanthera* (Stpiczyńska et al., 2005)], *Mentha piperita* (Amelunxen, 1965; Turner et al., 2000, 2012), *Perilla ocymoides* (Kashina and Danilova, 1993) and *Rosmarinus officinalis* (Böszörményi et al., 2020). Some authors suggested that light may play a role in the formation of these peculiar membrane structures in secretory cells (Kashina and Danilova, 1993), while others observed identical structures in the neck cells of the peltate glandular hairs of both light-grown and dark-forced shoots (Böszörményi et al., 2020). Detailed comparative analyses about these peculiar non-bilayer membranes of *Rosmarinus officinalis* plastids have clearly demonstrated important structural differences. The PLBs of etioplasts represented a highly ordered and symmetrical membrane organization when compared with the loose and irregular tubuloreticular membranes of the secretory plastids (Figure 5) (Böszörményi et al., 2020).

The PLB should be thus considered as a highly regular subtype of tubular complexes, in which membrane tubules are arranged into tetrahedral (Figure 4) or hexapodel units, which are then joined into a paracrystalline 3D spatial network of membranes (Figure 5) (Gunning, 1965, 2001; Solymosi and Schoefs, 2008; Rudowska et al., 2012; Solymosi and Aronsson, 2013; Kowalewska et al., 2019; Bykowski et al., 2020). The different PLB types observed in different species and plastids, at different stages of development are beyond the scope of this review.

In spite of their widespread occurrence, it is still unclear whether tubuloreticular membranes have any specific function (e.g., in the production of secreted compounds of plastids or storage or accumulation of some metabolites) or they simply reflect some disturbance in the membrane homeostasis. The latter possibility may be outlined by the fact that such structures in animals or humans are mostly associated with stressful or diseased conditions [in case of AIDS tubuloreticular membranes have even been suggested to represent an ultrastructural pathological marker of the disease (Almsherqi et al., 2006)] and may be induced by exogenous or endogenous interferon (Grimley et al., 1985; Feldman et al., 1986; Orenstein et al., 1987), halogenated pyrimidines (Hulanicka et al., 1977) or even pyridoxine deficiency (Datsis, 1993). Stressful conditions such as starvation (Chong et al., 2017) or UV-irradiation (Kovács and Keresztes, 2002) also led to the formation of tubuloreticular complexes in amoeba mitochondria and apple plastids, respectively. On the other hand, some works suggest that such structures may be involved in regeneration processes of endothelial cells in wounded tissues (Eady and Odland, 1975), or in viral DNA or RNA uptake procedures (Almsherqi et al., 2006; Almsherqi, 2010).

In case of the PLBs, several data clearly indicate that due to their very high surface-to-volume ratio, these membranes represent a kind of membrane depot from which the photosynthetic membranes of the chloroplasts can be formed very fast upon illumination (reviewed in Solymosi and Schoefs, 2010; Solymosi and Aronsson, 2013). Thus, the etioplast-to-chloroplast transformation is much faster than the proplastid-to-chloroplast transformation pathway (Liebers et al., 2017), because during the latter important *de novo* synthesis of membrane lipids is required in parallel with protein and pigment biosynthesis. In addition, the PLBs and the proper organization and oligomerization of LPOR as well as the presence of carotenoids clearly play a role in photoprotection of the porphyrin pigments upon illumination, while in the absence of them, often photooxidative stress is induced (Sperling et al., 1997; Erdei et al., 2005; Hideg et al., 2010; reviewed by Solymosi and Schoefs, 2010). Similarly, cubic membranes efficiently prevented lipid peroxidation and RNA damage under oxidative stress conditions (Almsherqi, 2010).

There is no consensus on the factors inducing the formation of tubuloreticular membrane organization. Some authors suggested that they are the result of altered lipid (cholesterol) homeostasis, lipid and fatty acid composition induced by for instance viral infection (Almsherqi, 2010; Deng et al., 2010), others suggested that they may be associated with altered carotenoid composition (Park et al., 2002; Cuttriss et al., 2007), membrane symmetry (Larsson and Larsson, 2014), special protein-protein interactions or lipid-to-protein ratio (Almsherqi et al., 2009) or low divalent ion concentrations (Almsherqi et al., 2006; Brasnett et al., 2017). Such membrane organization was also observed in the aqueous lipid-protein film of lung surfactants (Larsson and Larsson, 2014). Therefore, it might be possible that a kind of specific water-lipid-protein composition is responsible for the formation of such structures. Similarly, we may speculate that the accumulation of lipophilic compounds (isoprenoids, terpenes, carotenoids, fatty acids, etc.) within the membranes could be involved in the formation of the PLBs. In the next section, we'll briefly discuss data about factors influencing PLB structure in etioplasts.

#### The Role of Lipid-Protein Interactions in the Formation of the Prolamellar Bodies

The lipid composition of the PLBs of etioplasts and chloroplast thylakoids is basically similar (Selstam and Sandelius, 1984; Selstam, 1998; Fujii et al., 2018, 2019a,b; Yu et al., 2020) with slightly lower amount of unsaturated (18:3) fatty acids being present in the PLB membranes than in the thylakoids (Selstam and Sandelius, 1984; Selstam, 1998). The ratio of the non-bilayer lipid MGDG to DGDG is slightly higher (1.6–1.8) in purified PLBs and in chloroplast thylakoids (1.7) than in prothylakoids (1.1–1.4) (Ryberg et al., 1983; Sandelius and Selstam, 1984; Selstam and Sandelius, 1984). Data on chloroplast structure of different lipid biosynthesis mutants revealed that minor alterations in the MGDG:DGDG ratio may have important effect on plastid structure (Mazur et al., 2019; Yu et al., 2020) and that dynamic local changes of the neutral/anionic lipid ratios may have important role in thylakoid arrangement (Kobayashi and Wada, 2016).

It is also evident from studies using lipid biosynthesis mutants (Fujii et al., 2017) and from *in vitro* reconstitution experiments (Gabruk et al., 2017) that some lipids (MGDG, DGDG and SQDG) are crucial for the proper assembly of PLBs and also for their transformation into thylakoids upon illumination (Fujii et al., 2018). Fujii et al. (2019b) have reviewed in detail the role of different plastid lipids on the Mg-branch of porphyrin biosynthesis, therefore, we prefer not to discuss these data in detail in this work.

In spite of the similar lipid composition, major differences are observed in the protein composition of chloroplast thylakoids and PLBs (Ryberg and Sundqvist, 1982a; Selstam and Sandelius, 1984; von Zychlinski et al., 2005; Blomqvist et al., 2008; Kanervo et al., 2008) with the latter containing much less proteins (and thus a relatively high lipid to protein ratio) and among them predominantly LPOR.

The lipid-dependent formation of the photoactive oligomers was proposed to be a mechanism inducing the formation of the PLBs (Gabruk et al., 2017), and correlations were found between PLB formation and proper lipid composition and accumulation, and proper amounts of LPOR in other works as well (reviewed in Fujii et al., 2019b). Similarly, a clear relationship was found between the formation and accumulation of LPOR oligomers (especially LPOR-A) and the occurrence and size of PLBs in etioplasts (Sperling et al., 1997; Franck et al., 2000; Frick et al., 2003; Paddock et al., 2010, 2012). Studies using *Arabidopsis thaliana* mutants showed that the amounts of LPOR-A and LPOR-B correlate with PLB size. Inhibition of LPOR-A expression led to reduced PLB size (Frick et al., 2003; Paddock et al., 2010, 2012), while its overexpression resulted in larger PLBs (Sperling et al., 1997; Franck et al., 2000; Paddock et al., 2010, 2012).

Similar data were observed in pea with overexpressed or antisense-LPOR (Seyedi et al., 2001). Furthermore, in organs with low LPOR levels, proplastids are present and PLBs are scarce or small [e.g., in non-leaf organs of pea (Böddi et al., 1994)]. Recent data on cyanobacterial LPOR indicated that it is present in dimerized form *in vivo* (Schneidewind et al., 2019) and its overexpression induced the formation of tubuloreticular membranes slightly resembling PLBs within the cells accumulating LPOR and Pchilde but being deficient in NADPH (Yamamoto et al., 2020).

Similarly, the disaggregation of the LPOR oligomers or the degradation of LPOR are also strongly associated with the disruption of the regular structure of the PLBs (e.g., Ryberg and Sundqvist, 1988) both under dark conditions (and induced by stress factors, e.g., Solymosi et al., 2006b) or during the light-induced greening of etiolated leaves, during which the PLB is fully reorganized and transformed into developing thylakoids (Kowalewska et al., 2016; reviewed in Solymosi and Schoefs, 2010; Solymosi and Aronsson, 2013; Kowalewska et al., 2019). These large-scale membrane reorganizations occur in parallel with important spectral changes (e.g., the Shibata shift—Figure 3, Shibata, 1957) which reflect the disaggregation of Chlide pigments and further outline the strong structural connection between LPOR macrodomains and PLB membranes. The Shibata shift is strongly inhibited by low temperature, high pressure as well as protein cross-linkers, or glycerol and sugars (Wiktorsson et al., 1993; Zhong et al., 1996; Solymosi et al., 2002, 2007a; Smeller et al., 2003), i.e., factors and conditions which stabilize the oligomeric structure, inhibit its conformational changes, disaggregation or the dissociation of peripheral proteins from membranes. However, under normal conditions, in parallel with the disruption of the PLB structure, the LPOR oligomers also undergo disaggregation and release Chlide from their active site. After this, the photoactive LPOR oligomers are again regenerated by binding Pchlide (Granick and Gassman, 1970; Amirjani and Sundqvist, 2004; Rassadina et al., 2004; Schoefs and Franck, 2008) from the pool of non-photoactive Pchlide molecules, and they again catalyze Pchlide photoreduction in them. Several authors reported that until Chl biosynthesis is still active in organs, because their final Chl content has not been reached, PLBs (Ikeda, 1970, 1971) and LPOR oligomers persist in their plastids and may even accumulate under special light conditions (e.g., Solymosi et al., 2006a,b; Schoefs and Franck, 2008; Solymosi et al., 2012). The plastids of such low-light-grown or partially etiolated or young tissues contain simultaneously developing grana and PLBs, thus an important spatial and structural heterogeneity can be clearly observed in the organization of the plastid inner membranes in these so-called etio-chloroplasts. Unfortunately, the PLBs of etio-chloroplasts seem to be versatile structures hard to isolate, therefore, we have not much information about the lipid and protein composition of these membranes.

## Concluding Remarks

Most data on Chl biosynthesis and especially about Pchlide photoreduction and its molecular details were obtained using etiolated seedlings. The use of etiolated systems to study these processes were often criticized because the major chloroplast differentiation pathway under natural light conditions is the proplastid-to-chloroplast pathway occurring in most seeds germinating on the soil surface or in new leaves produced by the shoot apical meristem (Charuvi et al., 2012; Yadav et al., 2019). However, several data indicate that etioplasts and accumulation of Pchlide and LPOR ternary complexes occur also under natural conditions in seedlings germinating in the soil (e.g., Vitányi et al., 2013; Kakuszi et al., 2017), in tissues partially covered by other organs (e.g., Solymosi et al., 2007b), in inner leaf primordia developing inside closed bud structures (e.g., Solymosi et al., 2004, 2006a, 2012), and in other systems (water plants, etc., reviewed in Solymosi and Aronsson, 2013; Armarego-Marriott et al., 2020). In such tissues or organs Chl biosynthesis and etioplast-to-chloroplast transformation may be similar to those described in completely etiolated seedlings, but the processes and their regulation still need to be elucidated in such naturally etiolated tissues.

In addition, photosynthesis requires a constant supply of Chls especially in plants under fluctuating or stressful conditions. Several data indicate that for instance after recovery from desiccation or drought stress (e.g., Solymosi et al., 2013; Liu et al., 2018, 2020), Chl synthesis genes are upregulated and an intensive biosynthesis occurs. So far only limited amount of information is available about LPOR catalytic activity and macromolecular organization in chloroplasts. Taken together, a better understanding of the Mg-branch of Chl biosynthesis, especially of LPOR activity, structure and location, and factors involved in its regulation in chloroplasts and etio-chloroplasts of various green (crop) plants may be also important for agriculture. The recent breakthroughs in molecular biology (e.g., next generation sequencing and tremendous developments in various omics techniques which enable the identification of different isoforms and their expression and translational patterns in various plant species and plastid subcompartments) and microscopic methods, as well as the use of various novel model systems (i.e., dark-forced tobacco shoots—Armarego-Marriott et al., 2019, duckweed—Monselise et al., 2015) will probably provide novel and interesting data on the exact molecular regulation of these processes and the role of lipids in them.

A large step into the understanding of LPOR-lipid interactions has been achieved by the *in vitro* reconstitution of photoactive LPOR macrodomains in the presence of lipids (Nguyen et al., 2021). These data demonstrate for the first time directly the role of LPOR and LPOR-lipid interactions in the formation of a special membrane phase and helical organization. In addition to analyses on different mutants, similar *in vitro* reconstitution experiments and membrane and lipid binding assays of various LPOR isoforms as well as other Chl biosynthesis enzymes of different species should be performed to increase our understanding of the membrane association and localization of Chl biosynthesis. It is also important to outline, that at the moment our data are mostly related to the 3 LPOR isoforms of Arabidopsis, and the 2 isoforms present in few crop species (rice, barley), but further experiments with other crops with less or more isoforms are necessary to understand LPOR activity and its universal or specific regulation in case of the various isoforms and species. The same applies to other enzymes of Chl biosynthesis. Due to its major and central role in autotrophic plant metabolism, we believe that an increased understanding of Chl biosynthesis may be crucial to breed plants with improved quality, e.g., higher yield or performance under adverse environmental conditions.

## Author Contributions

KS and BM-K equally contributed to the conceptualization, writing, and discussion about the manuscript's content. Both authors edited and approved the submitted version.

## Conflict of Interest

The authors declare that the research was conducted in the absence of any commercial or financial relationships that could be construed as a potential conflict of interest.

## Abbreviations

Chl: chlorophyll
Chlide: chlorophyllide
DPOR: dark-operative NADPH:Pchlide oxidoreductase enzyme
DGDG: digalactosyldiacylglycerol
DV-Pchlide: divinyl-Pchlide (3;8 vinyl-Pchlide)
DVR: divinyl reductase
FNR1: ferredoxin-NADPH reductase 1
GG-Chlide: geranylgeranyl chlorophyllide
LPOR: light-dependent NADPH:protochlorophyllide oxidoreductase
MEP: 2-methylerythritol-4-phosphate pathway
MGDG: monogalactosyldiacylglycerol
MV-Pchlide: protochlorophyllide
Pchlide: protochlorophyllide
PG: phosphatidylglycerol
PLB: prolamellar body
PTs: prothylakoids
SQDG: sulfoquinovosyldiacylglycerol.
